# Adherence to the Dietary Index for Gut Microbiota and reduced risk of nephrolithiasis: a case–control study of Chinese adults

**DOI:** 10.3389/fnut.2026.1893492

**Published:** 2026-09-04

**Authors:** Xing Jin, Jia Li, Yuanqi Zhao, Kun Xu, Yuge Wang, Song Bai, Yashu Liu, Zhenhua Li

**Affiliations:** 1 Department of Nursing, Shengjing Hospital of China Medical University, Shenyang, Liaoning, China; 2 Department of General Surgery, Shengjing Hospital of China Medical University, Shenyang, China; 3 China Medical University–The Queen's University of Belfast Joint College, Shenyang, China; 4 Department of Urology, Shengjing Hospital of China Medical University, Shenyang, China; 5 Department of Pathology, Shengjing Hospital of China Medical University, Shenyang, China

**Keywords:** case–control study, Dietary Index for Gut Microbiota, DI-GM, gut microbiota, nephrolithiasis

## Abstract

**Objective:**

Nephrolithiasis represents a substantial clinical burden worldwide. The Dietary Index for Gut Microbiota (DI-GM) assesses dietary impacts on gut microbiota. However, its association with the risk of nephrolithiasis remains unclear in Chinese adults.

**Methods:**

We conducted a case–control study using the Shenyang sub-cohort of the Tianjin Chronic Low-Grade Systemic Inflammation and Health (TCLSIH) cohort. Cases were 278 patients with nephrolithiasis. Controls were 556 healthy individuals, matched 1:2 by age and sex. Dietary intake was assessed using a validated food frequency questionnaire (FFQ) to calculate DI-GM scores. Multivariable conditional logistic regression evaluated the association. Restricted cubic spline (RCS) explored non-linear associations, and subgroup analyses were stratified by age and sex.

**Results:**

Higher DI-GM scores were significantly associated with lower risk of nephrolithiasis. After adjustment, the risk decreased progressively with increasing DI-GM scores (P for trend < 0.05). Individuals with DI-GM ≥ 8 had 83% lower risk than those scoring 0–5 (odds ratio [OR] = 0.17, 95% confidence interval [CI]: 0.08–0.36). The association was consistent across age and sex subgroups, with no non-linearity (P for non-linearity > 0.05).

**Conclusion:**

In Chinese adults, higher adherence to the DI-GM was associated with lower risk of nephrolithiasis. This finding suggests gut-friendly dietary patterns may be associated with a lower likelihood of nephrolithiasis. Future prospective studies are warranted to confirm this association.

## Introduction

Nephrolithiasis is a global public health concern due to its high prevalence and high recurrence rate. The incidence of the disease is increasing markedly in many regions (1–4). Without active medical management, the 10-year recurrence rate exceeds 50% (5). Clinically, nephrolithiasis ranges from asymptomatic cases to severe manifestations, including renal colic, hematuria, acute kidney injury, and chronic kidney disease (6–8). The rate of hospitalization for surgical intervention is as high as 26% (8), imposing a substantial economic and social burden (9). Therefore, identifying factors associated with nephrolithiasis is of clinical importance.

Gut microbiota may influence nephrolithiasis pathogenesis through several mechanisms, including the regulation of intestinal oxalate absorption, uric acid homeostasis, lipid metabolism, and inflammatory responses (10, 11). Diet is a key determinant of gut microbiota composition, function, and diversity (12, 13). The Dietary Index for Gut Microbiota (DI-GM) was developed based on a systematic literature review to assess dietary patterns that may benefit or harm gut microbiota. The index comprises 10 beneficial components (e.g., dietary fiber, fermented dairy) and 4 detrimental components. A composite score is calculated to reflect the overall dietary influence on gut microbiota (14, 15). Therefore, the DI-GM score, which reflects dietary patterns and their impact on gut microbiota, may help identify individuals with a lower likelihood of developing nephrolithiasis.

Prior studies using NHANES data have examined the association between DI-GM and the risk of nephrolithiasis (16–18). One study reported a significant linear inverse association (16), whereas two others identified nonlinear inverse associations (17, 18), indicating that this relationship remains unresolved. Notably, nephrolithiasis diagnosis in these studies relied on self-administered questionnaires, which raises concerns about potential misclassification bias. Furthermore, existing evidence is derived predominantly from the US NHANES, which captures dietary patterns substantially different from those in Asia, particularly China (19). For example, Asian dietary patterns differ substantially from Western diets, typically characterized by higher carbohydrate and lower fat intake (20). Socioeconomic status varies considerably between Asian and Western populations (21), and both dietary patterns and socioeconomic status are known to influence the risk of nephrolithiasis (22, 23). To our knowledge, although the association between DI-GM and the risk of nephrolithiasis has been investigated in US-based studies (16–18), no study has specifically examined this association in a Chinese population. Furthermore, patients with nephrolithiasis face higher risks of severe complications, such as sepsis (24).

Therefore, it is necessary to further investigate the association between DI-GM and the risk of nephrolithiasis in Asian populations.

## Materials and methods

### Participants

This study used data from the Tianjin Chronic Low-Grade Systemic Inflammation and Health (TCLSIH) cohort, a large-scale prospective dynamic cohort established in Tianjin, China, in 2007 (25). The design and data collection procedures of the TCLSIH cohort have been described in detail elsewhere (26). The cohort is characterized by its unique focus on chronic low-grade inflammation, large sample size, prospective design, and high-quality multidimensional data, making it a valuable platform for investigating the etiology of chronic diseases and advancing precision prevention in the Chinese population. Since 2019, the TCLSIH cohort has expanded into a multi-center study, with sub-cohorts established in nine Chinese cities, including Shenyang. Both the nephrolithiasis cases and the healthy controls in the present study were derived from the Shenyang sub-cohort.

**Figure 1:**
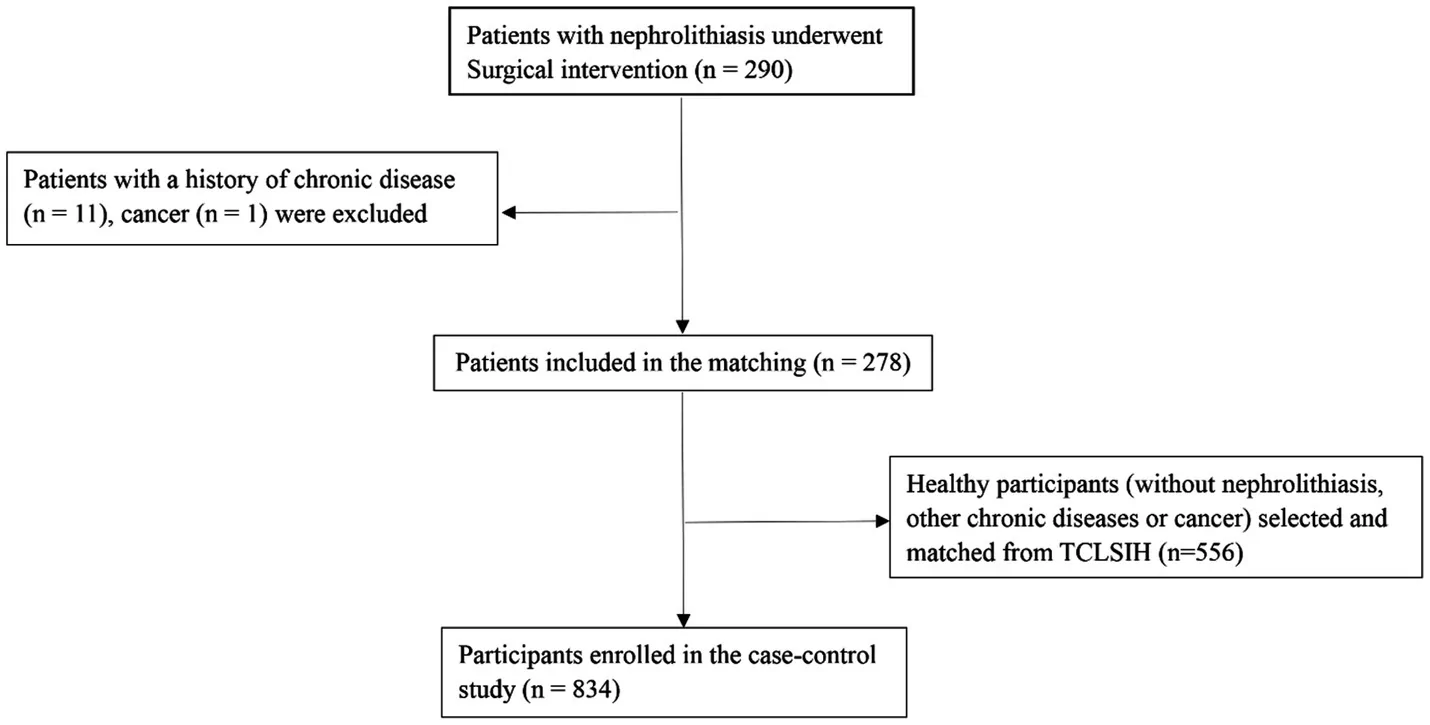
Flowchart.

Cases were participants from this sub-cohort who were newly diagnosed with nephrolithiasis and who required surgical intervention at the Department of Urology, Shengjing Hospital of China Medical University, between 2019 and 2021. After excluding 11 patients with any chronic disease and one patient with cancer, 278 eligible patients were included in the final analysis. Controls were selected from the same sub-cohort participants who remained free of nephrolithiasis during the same follow-up period (2019–2021), matched 1:2 to cases by age (± 1 year) and sex, and had no history of nephrolithiasis, other chronic diseases, or cancer. Thus, this case–control study ultimately included 278 nephrolithiasis cases and 556 healthy controls, totaling 834 participants (Figure 1).

The study protocol was approved by the Ethics Committee of Shengjing Hospital of China Medical University (2019PS679K). The study was conducted in accordance with the ethical principles of the Declaration of Helsinki (1975). All participants provided written informed consent after being fully informed of the study objectives, procedures, and potential risks.

### Assessment of nephrolithiasis

All patients with nephrolithiasis underwent surgical intervention, including retrograde intrarenal surgery (RIRS), shock wave lithotripsy (SWL), or percutaneous nephrolithotomy (PNL). Case identification was based on the ICD-10 code N20, recorded as either the primary or secondary diagnosis. When N20 was recorded as a secondary diagnosis, patients had been admitted for other conditions but concurrently received surgical intervention for nephrolithiasis due to acute stone-related symptoms (e.g., renal colic, urinary obstruction) or for prophylactic indications.

### Assessment of the DI-GM

Information on the intake of foods and beverages in the last month was assessed using a validated 100-item food frequency questionnaire (FFQ) (27, 28) with specified serving sizes. For each food item and beverage, consumption frequency was evaluated using a seven-point scale (ranging from “almost never” to “≥ 2 times/day”) and an eight-point scale (ranging from “almost never” to “≥ 4 times/day”), respectively. Daily intake of each food item (grams/day) was calculated by multiplying the portion size per eating occasion by the daily consumption frequency. Nutrient intake from each food was then estimated by multiplying its daily intake amount by its nutrient content per 100 g, according to the Chinese Food Composition Tables (29). Total daily nutrient intake was obtained by summing these values across all food items. To evaluate the potential impact of diet on gut microbiota, we used the DI-GM. This index comprises 14 dietary components: 10 beneficial components (avocado, broccoli, chickpeas, coffee, cranberry, fermented dairy, dietary fiber, green tea, soy, and whole grains) and 4 detrimental components (red meat, processed meat, refined grains, and high-fat diet). Scoring was performed as follows. For beneficial components, a score of 1 was assigned if intake was above the sex-specific median intake of the study population; otherwise, 0. For detrimental components, a score of 1 was assigned if intake was below the sex-specific median; otherwise, 0. The scores for all components were summed to generate the total DI-GM score, which theoretically ranges from 0 to 14 (Supplementary Table 1). A higher total score indicates a dietary pattern associated with favorable gut microbiota-related characteristics (14, 15).

### Assessment of covariates

Detailed information for all participants was collected using structured questionnaires. Anthropometric parameters, including height and weight, were measured following standardized protocols. Body mass index (BMI) was calculated as weight in kilograms divided by height in meters squared (30). Sociodemographic variables included age, educational attainment (categorized as less than college graduate, college graduate, or postgraduate), and income. Behavioral variables included smoking status (current, former, or never smoker), alcohol consumption status (current, former, or never drinker), daily water intake (measured in 250 mL cups), and total energy intake. Total daily energy intake (kilocalories) was calculated by summing the energy contribution of all consumed food items, derived from their respective daily intake amounts and energy density.

### Statistical analysis

Baseline characteristics of patients with nephrolithiasis and healthy controls were described. Categorical variables were presented as frequencies and percentages and compared using the chi-square test. Continuous variables were expressed as least-squares means with 95% confidence intervals (CIs) and compared using Student’s *t*-test, with consideration of their distributions. To examine the association between the DI-GM and the risk of nephrolithiasis, conditional logistic regression was used, and the strength of the association was quantified using odds ratios (ORs) with corresponding 95% CIs. The linear trend across the three groups (based on the distribution of DI-GM scores) was tested by entering the median value of each group as a continuous variable in the conditional logistic regression. Model 1 was the crude model with no adjustments. Model 2 was adjusted for body mass index (BMI). Model 3 was further adjusted for income, educational attainment, smoking status, alcohol consumption, daily water intake, and total energy intake. Participants with unknown values for categorical covariates, such as smoking and drinking status, were classified into a separate category and incorporated into the conditional logistic regression models. To assess potential non-linear associations between the DI-GM score and the risk of nephrolithiasis, restricted cubic spline (RCS) analysis was performed using the fully adjusted model, with three knots placed at the 25th, 50th, and 75th percentiles of the DI-GM score and the lowest score set as the reference. Subgroup analyses were performed stratified by age and sex to evaluate the influence of potential confounders. All statistical analyses were performed using SAS version 9.3 for Windows (SAS Institute Inc., Cary, NC, USA). A two-sided *p* value < 0.05 was considered statistically significant.

## Results

### Baseline characteristics of the study population

Table 1 presents the baseline sociodemographic characteristics, anthropometric measures, behavioral habits, and dietary patterns of patients with nephrolithiasis and healthy controls. Compared with healthy controls, these patients had a higher prevalence of smoking and alcohol consumption, as well as a higher proportion of water intake ≥ 1,200 mL; conversely, they had lower BMI, income, educational attainment, and total energy intake.

**Table 1:** Characteristics of the nephrolithiasis patients and healthy controls.

Characteristics	Patients	Controls	*p* ^a^
	*n* = 278	*n* = 556	
Age (years)	53.79 (52.29, 55.29)^b^	53.91 (52.85, 54.97)	0.90
Sex (male, %)	185 (66.55)	372 (66.91)	0.92
Body mass index (kg/m^2^)	23.96 (23.54, 24.37)	25.49 (25.20, 25.79)	**<0.0001**
Income (≥10,000 Yuan per month, %)	19 (6.83)	173 (31.12)	**<0.0001**
Education (college graduate or higher, %)	42 (15.11)	235 (42.27)	**<0.0001**
Smoking status (%)			
Smoker	103 (37.05)	128 (23.02)	**<0.01**
Ex-smoker	21 (7.55)	61 (10.97)	0.06
Non-smoker	154 (55.40)	324 (58.27)	**0.03**
Unknown	0 (0.00)	43 (7.73)	**<0.0001**
Drinking status (%)			
Drinker	77 (27.70)	102 (18.35)	**<0.0001**
Ex-drinker	35 (12.59)	134 (24.10)	0.48
Non-drinker	164 (58.99)	303 (54.50)	**<0.0001**
Unknown	2 (0.72)	17 (3.06)	0.75
Water intake (≥1,200 mL/day, %)	101 (36.33)	183 (32.91)	**0.01**
Energy intake (kcal/day)	1,440.28 (1,359.22, 1,521.34)	1,712.41 (1,655.09, 1,769.73)	**<0.0001**

a Analysis of Student *t*-test or chi-squared test.

b Least-square mean (95% confidence interval).Bold *p* values indicate statistically significant differences (*p* < 0.05).

### Association between DI-GM and the risk of nephrolithiasis

**Figure 2:**
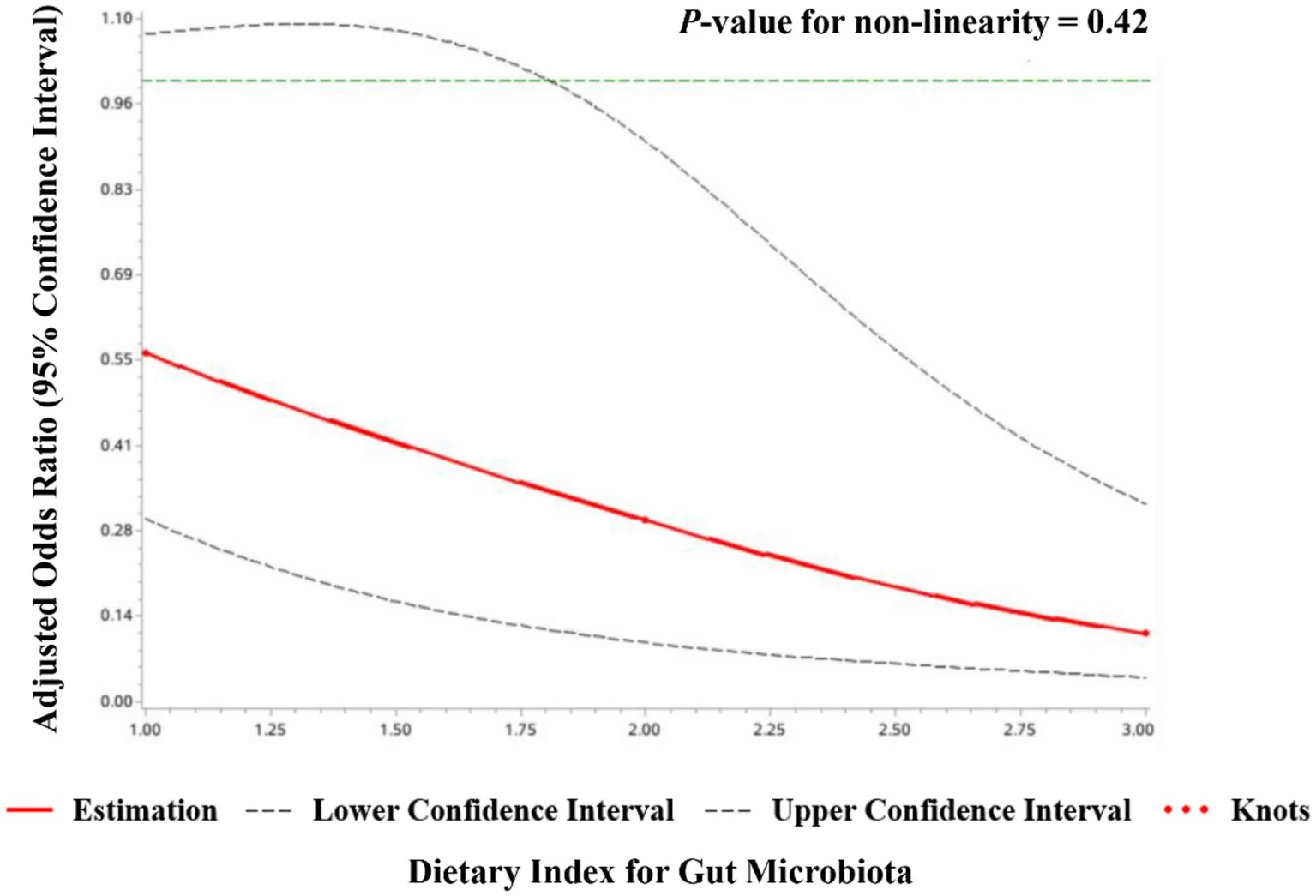
Restricted cubic splines for the associations between DI-GM and the risk of nephrolithiasis. Odds ratio and 95% confidence intervals were calculated by conditional logistic regression models adjusted for income, BMI, education, smoking status, drinking status, water intake and energy intake.

Table 2 presents the crude and multivariable-adjusted associations between the DI-GM score and the risk of nephrolithiasis. Categorical analysis of the DI-GM score revealed that a score of ≥ 8 was significantly inversely associated with this condition (crude OR = 0.18, 95% CI: 0.11–0.27; adjusted OR = 0.17, 95% CI: 0.08–0.36). Further analysis focusing on the beneficial components of the DI-GM showed that they were associated with lower risk of nephrolithiasis in both Model 2 and Model 3 (OR = 0.35, 95% CI: 0.25–0.50; and OR = 0.39, 95% CI: 0.23–0.65, respectively). Trend tests indicated a significant decreasing trend in the risk of this condition with increasing DI-GM score (*p* for trend < 0.05). RCS analysis found no evidence of a non-linear association between the DI-GM score and the risk of nephrolithiasis (*p* for non-linearity > 0.05) (Figure 2).

**Table 2:** Conditional logistic regression analysis of the association between DI-GM and the risk of nephrolithiasis.

Characteristic	Model 1^a^	Model 2^b^	Model 3^c^
DI-GM group			
0–5	Reference	Reference	Reference
6–7	0.41 (0.28, 0.60)^e^	0.45 (0.30, 0.67)	0.38 (0.20, 0.73)
≥8	0.18 (0.11, 0.27)	0.17 (0.11, 0.27)	0.17 (0.08, 0.36)
*p* for trend^d^	<0.0001	<0.0001	<0.0001
Unfavorable to gut microbiota			
<2	References	References	References
≥2	0.21 (0.15, 0.31)	0.20 (0.14, 0.30)	0.24 (0.13, 0.45)
Beneficial to gut microbiota			
<5	References	References	References
≥5	0.33 (0.23, 0.46)	0.35 (0.25, 0.50)	0.39 (0.23, 0.65)

DI-GM, Dietary Index for Gut Microbiota.

a Model 1 was crude model.

b Model 2 was adjusted for BMI.

c Model 3 was further adjusted for income, education, smoking status, drinking status, water intake, and energy intake based on model 2.

d Multivariate conditional logistic regression.

e Odds ratio (95% confidence interval) (all such values).

### Subgroup analysis

As shown in Table 3, a consistent inverse association between the DI-GM score and the risk of nephrolithiasis was observed across all age and sex subgroups. No significant interactions were detected between the DI-GM score and age or sex (*p* for interaction > 0.05).

**Table 3:** Subgroup analysis of the association between DI-GM and the risk of nephrolithiasis.

Subgroup	DI-GM group			*p* for interaction
	0–5	6–7	≥8	
Age				0.83
≤60	Reference	0.36 (0.16, 0.81)^a^	0.15 (0.06, 0.38)	
>60	Reference	0.33 (0.07, 1.50)	0.13 (0.02, 0.93)	
Sex				0.93
Female	Reference	0.39 (0.17, 0.90)	0.18 (0.07, 0.45)	
Male	Reference	0.32 (0.08, 1.21)	0.06 (0.01, 0.37)	

DI-GM, Dietary Index for Gut Microbiota.

a Odds ratio (95% confidence interval) (all such values). Odds ratio and 95% confidence intervals were calculated by conditional logistic regression models adjusted for age, income, BMI, education, smoking status, drinking status, water intake, and energy intake (age was not adjusted in age subgroup analysis).

## Discussion

In this case–control study, a clear inverse association was observed between the DI-GM score and the risk of nephrolithiasis. Participants with a DI-GM score ≥ 8 had 82% lower risk of nephrolithiasis compared with those with lower scores. This inverse association remained robust after extensive adjustment for multiple confounders. Subgroup analyses confirmed the consistency of this association across sex and age groups.

Three prior cross-sectional studies using the NHANES database have investigated the association between the DI-GM and the risk of nephrolithiasis (16–18), but their findings were not entirely consistent. One study using data from 2007 to 2020 reported a significant linear inverse association between the DI-GM and the risk of nephrolithiasis (16). In contrast, two other studies, despite using data from overlapping periods but with different sample sizes, reported a non-linear association (17, 18). These differences may be attributable to the use of self-reported nephrolithiasis history as the outcome definition, which is subject to recall bias. In the current study, case identification was based on ICD-10 code N20 and confirmed through systematic review of hospital surgical records. This approach allowed for objective case ascertainment and effectively reduced recall bias, offering an advantage over traditional questionnaire-based surveys. As noted in previous studies, the lack of data on specific tea types in the NHANES database precluded the inclusion of green tea in the DI-GM scoring, which may have introduced constraints in those prior analyses. In addition, differences in participant characteristics across studies, such as dietary habits, genetic predispositions, and geographical variations, may have influenced the results.

The DI-GM is a literature-derived dietary index associated with gut microbiota (14, 15). In this case–control study, we observed an inverse association between the DI-GM and the risk of nephrolithiasis. Although we did not directly measure gut microbiota, previous literature suggests several mechanisms that could explain this association. First, certain gut microbiota may influence stone formation by modulating the metabolism of key substances. For example, oxalate-degrading bacteria, such as *Oxalobacter formigenes* and certain strains of Bifidobacterium and Lactobacillus, can degrade intestinal oxalate, reducing its absorption and consequently lowering urinary oxalate levels, which may be associated with a lower risk of calcium oxalate stones (31–34). Conversely, bacteria such as Klebsiella species can convert melamine to cyanuric acid, potentially inducing nephrotoxic stones (35). Second, metabolites produced by gut microbiota, such as short-chain fatty acids (e.g., butyrate and acetate), may enhance intestinal barrier function and modulate systemic inflammation and oxidative stress. These metabolites may contribute to renal health by influencing citrate excretion and urine pH, which may be associated with lower risk of nephrolithiasis (36–39). These findings suggest that modulating dietary patterns to support gut microbiota, and utilizing assessment tools such as the DI-GM to reflect dietary influences on gut ecology, could be a potential focus for future research on nephrolithiasis.

Previous investigations have established that patients with nephrolithiasis exhibit marked gut microbiota dysbiosis relative to healthy controls (40). Notably, patients with recurrent disease who demonstrate an elevated propensity for surgical intervention present a more severe dysbiotic profile, with further reduction of beneficial bacteria and enrichment of pro-inflammatory taxa (41). This observed gradient is consistent with our findings, supporting the inference that a higher DI-GM score is associated with reduced risk of classification into the most severe nephrolithiasis subgroup, which harbors the greatest degree of microbial disruption.

Our study found that higher adherence to the DI-GM was associated with reduced risk of nephrolithiasis, suggesting the potential utility of gut microbiota-targeted dietary patterns in the risk of nephrolithiasis. The gut-kidney axis has been proposed as a key mechanism in nephrolithiasis pathogenesis. Previous studies have suggested that this axis is a central driver of nephrolithiasis, with stone formers exhibiting gut dysbiosis characterized by reduced oxalate-degrading bacteria and enrichment of pro-inflammatory taxa (42). Genetic factors, treatment modalities, gut microbiota, and artificial intelligence applications warrant further investigation. Genetic factors contribute to the development and progression of nephrolithiasis. For instance, susceptibility to urolithiasis has been associated with the TaqI genotype, with the Tt and tt genotypes potentially increasing the incidence of urolithiasis and the TT genotype conferring a reduced risk (43). Furthermore, genetic susceptibility may interact with lifestyle factors in the association with nephrolithiasis (44). Advances in treatment modalities also warrant further investigation. For example, one study demonstrated that, relative to the holmium-yttrium-aluminum-garnet laser, thulium fiber laser lithotripsy offered superior efficacy, as evidenced by a higher stone-free rate, shorter operative and lasing times, and a better intraoperative safety profile, including fewer complications and shorter hospital stay, although it carried a higher risk of postoperative sepsis (45). Another study examined the association between cinacalcet combined with sevelamer hydrochloride and urolithiasis in maintenance hemodialysis patients from a pharmacological standpoint. The findings indicated that, compared with sevelamer hydrochloride alone, the combined therapy significantly reduced the incidence and recurrence rate of urolithiasis, inhibited stone enlargement, and regulated urine pH, thereby contributing to the prevention and management of urolithiasis in patients with diabetic kidney disease (46). The impact of the COVID-19 pandemic on the diagnosis-to-treatment interval and treatment outcomes of nephrolithiasis is also noteworthy. Previous studies have reported that during the pandemic, complication rates and diagnosis-to-treatment intervals were significantly higher in patients with renal and ureteral stones compared with the pre-pandemic period (47). Finally, with the advancement of artificial intelligence, future research should promote the application of large language models in urolithiasis management, enhancing their precision in handling complex clinical scenarios and ensuring the transparency and verifiability of their outputs (48).

This study has several strengths. First, to our knowledge, it is the first to investigate the association between the DI-GM and the risk of nephrolithiasis in an Asian population, addressing a previous gap in evidence from non-US populations. Second, the database used in this study included all 14 dietary components required to calculate the DI-GM, ensuring the completeness and accuracy of the index computation and thereby strengthening the scientific validity of the findings. Third, case identification was based on ICD-10 code N20 and confirmed through systematic review of hospital surgical records. This approach circumvented the recall bias commonly associated with traditional questionnaire-based studies, enhancing the reliability of the conclusions. Overall, this study provides empirical evidence supporting an association between the DI-GM and the risk of nephrolithiasis in Chinese adults.

This study also has several limitations. First, the DI-GM was calculated based on FFQ, which may be subject to recall bias. Second, the DI-GM is an indirect proxy for gut microbial composition rather than a direct measure derived from microbiome sequencing. Therefore, we cannot confirm that the observed association between DI-GM and the risk of nephrolithiasis is indeed mediated through the gut microbiota. Our mechanistic discussion, while informed by prior literature, remains speculative without direct microbiome data. Future studies incorporating metagenomic or 16S rRNA sequencing are essential to validate the presumed mediating role of the gut microbiome and to establish causality. Third, the case–control design precludes causal inference regarding the relationship between the DI-GM and the risk of nephrolithiasis and cannot assess long-term effects. Fourth, the cases in this study were limited to patients with nephrolithiasis who required surgical intervention. Although this approach enhances diagnostic certainty and clinical relevance, it may introduce selection bias. Caution is therefore warranted when generalizing our findings to all individuals with nephrolithiasis, particularly those with asymptomatic or conservatively managed nephrolithiasis. Fifth, although dietary habits are generally recognized as relatively stable over time (49), it is not possible to completely exclude reverse causation with a case–control study design. Future prospective cohort studies are warranted to further investigate the aforementioned association. Sixth, although our study was designed to examine the association between adherence to the DI-GM and the risk of nephrolithiasis, independent of stone-related information or the specific surgical procedure, we did not report data on stone composition, location, burden, or the distribution of surgical procedures. This limits the ability to assess whether the association varies by stone-related information or surgical procedure type. Seventh, data on urate levels, urinary oxalate excretion, other related biomarkers and additional residual confounding factors were not obtainable because these measures are not included in routine clinical examinations. We were therefore unable to conduct sensitivity analyses to assess the potential influence of these variables. Eighth, patients with nephrolithiasis had lower BMI than healthy controls. Although lower total energy intake may partly account for this observation, other factors cannot be excluded, including selection bias from enrolling cases who required surgical intervention, the possibility of dietary changes, and residual confounding from unmeasured variables. Future prospective cohort studies are warranted to further investigate the underlying reasons for this BMI difference. Finally, although the models adjusted for demographic characteristics, lifestyle factors, and common comorbidities, we cannot entirely rule out the influence of other unmeasured confounding factors, such as gastrointestinal tract diseases, some neurogenic diseases, family history of nephrolithiasis, urinary citrate levels, urinary oxalate excretion, serum calcium, uric acid levels, and use of certain medications (e.g., diuretics, calcium supplements, and vitamin D). In conclusion, future large-scale prospective cohort studies are warranted to elucidate the underlying mechanisms and potential causal relationships between the DI-GM and the development of nephrolithiasis.

## Conclusion

In this case–control study of Chinese adults, higher adherence to the DI-GM was associated with lower risk of nephrolithiasis. This finding suggests that a gut-friendly dietary pattern may be associated with a lower likelihood of nephrolithiasis. Future studies are warranted to further explore the specific role of the gut microbiota in the pathogenesis of nephrolithiasis.

## Data Availability

The original contributions presented in the study are included in the article/Supplementary material, further inquiries can be directed to the corresponding authors.
