# Supplementary material for: Adherence to the Dietary Index for Gut Microbiota and reduced risk of nephrolithiasis: a case–control study of Chinese adults
Source: Front Nutr. 2026 Sep 4;13:1893492. doi: 10.3389/fnut.2026.1893492 (PMC13585611; doi:10.3389/fnut.2026.1893492)
Supplement: Supplementary file 1 [file Table_1.docx]

**Supplementary Table 1. Components of the DI-GM.**

| Component | Included Foods within the Component | Scoring |
| --- | --- | --- |
| Beneficial to gut microbiota |  | |
| Avocados | Avocados | For each component, a score of 1 if consumption at or above the sex-specific median, else 0 |
| Broccoli | Broccoli |  |
| Chickpea | Chickpeas |  |
| Coffee | Coffee |  |
| Cranberries | Cranberries |  |
| Fermented dairy | Yogurt, cheese, kefir, sour cream, buttermilk |  |
| Fiber | Not applicable |  |
| Green tea | Green tea |  |
| Soybean | Soy products—Soy milk, Tofu |  |
| Whole grains | Grains defined as whole grains, containing the entire grain kernel―the bran, germ, and endosperm |  |
| Unfavorable to gut microbiota |  | |
| High-fat diet (% energy) | Not applicable | 0 if consumption at or above 40% energy from fat, else 1 For each remaining component, a score of 0 if consumption at or above the sex-specific median, else 1 |
| Processed meat | Frankfurters, sausages, corned beef, and luncheon meat that are made from beef, pork, or poultry |  |
| Red meat | Beef, veal, pork, lamb, and game meat; excludes organ meat and cured meat |  |
| Refined grains | Refined grains that do not contain all of the components of the entire grain kernel |  |
